# Participation in Healthy Ageing Centres in Bosnia and Herzegovina Is Associated with Increased Physical Activity, Social Interactions, and Life Satisfaction Among Older People: A Cross-Sectional Study

**DOI:** 10.3390/jal5010005

**Published:** 2025-02-06

**Authors:** Daniela Pamias-Lopez, Tara Keck

**Affiliations:** Department of Neuroscience, Physiology and Pharmacology, University College London, 21 University Street, London WC1E 6DE, UK

**Keywords:** ageing, healthy behaviours, physical activity, loneliness, social engagement, quality of life, Bosnia and Herzegovina

## Abstract

**Background:**

The global population is experiencing a demographic shift towards older ages, which has the potential to increase the prevalence of ageing-related diseases and associated healthcare costs. Promoting healthy ageing behaviours, such as physical and social activity, has been shown to reduce disability and frailty among older people and improve their life satisfaction. To this aim, several Healthy Ageing Centres have been established across nine municipalities in Bosnia and Herzegovina to support healthy ageing behaviours in older populations. This cross-sectional study hypothesises that participation in these centres will be associated with an increase in healthy behaviours.

**Methods:**

This exploratory study compares the behaviours of Healthy Ageing Centre members (*n* = 399) and non-members (*n* = 55) to assess if participation in Healthy Ageing Centres is associated with healthy ageing behaviours such as physical activity, social interactions, and life satisfaction.

**Results:**

Members at Healthy Ageing Centres had a higher life satisfaction, exercised for significantly longer, and engaged in social activities more frequently than non-members. No differences were found in diet, alcohol consumption or loneliness levels.

**Conclusions:**

The present study highlights the positive behaviours associated with attending Healthy Ageing Centres, suggesting that their establishment in ageing populations could be beneficial for supporting healthy ageing.

## 1 Introduction

There is a global trend toward population ageing, with the proportion of the population over 65 years old expected to increase by over 60% by 2050 [1]. In countries in Eastern Europe and Central Asia, including Bosnia and Herzegovina (BiH), this demographic shift is particularly strong due to a combination of long life expectancy, low birth rates, and the high emigration levels of young working citizens to Western Europe. As of 2023, 18% of the total population of BiH was over the age of 65, an increase of 11 percentage points in the last 30 years [2], which is consistent with the types of changes observed in many other countries in the Eastern Europe and Central Asia region [2]. While longevity provides opportunities for intergenerational learning and relationship building, it is also a risk factor for several chronic diseases including cancer, diabetes, cardiovascular disease, and dementia [3], with almost 95% of people over 65 years old experiencing at least one chronic condition [4]. Despite there being an understandably large focus on the management of ageing-related diseases [5], encouraging preventative strategies to support healthy ageing has the potential to extend life expectancy [6], delay the onset of ageing-related diseases, and potentially reduce the risk of developing these conditions all together [7].

One of the most effective ways to prevent ageing-related diseases is to promote healthy ageing lifestyle behaviours [6,8], including regular physical activity [9], social activities [10], a healthy diet [11], a reduction in smoking and alcohol intake [12], and regular sleep [13]. Physical activity is correlated with a reduction in mortality, disability, and frailty in older people, as well as improved mental function [14,15]. As frailty is associated with a wide range of medical conditions and comorbidities, including depression [16], heart disease [17,18], fractures, and general adverse health outcomes [16], it is particularly important to address in older populations [19]. Physical activity has also been shown to reverse the effects of several chronic conditions, even in people who only become physically active in later life [9], highlighting its potential for supporting healthy ageing throughout the lifespan. Physical activity can also reduce obesity and protect against insulin resistance, an established risk factor for many ageing-related diseases [20,21].

While physical activity has many benefits, quantifying its intensity can be challenging. As a result, measures of the metabolic equivalent of task (METs) are often used to quantify the intensity of different physical activities [22], with MET hours providing an integrated measure of total physical activity time and intensity. Engaging in a higher number of MET hours per week is associated with a decrease in chronic diseases, mobility impairment, and risk for several diseases, as well as an increase in good mental health, cognitive function, and social engagement [23].

Several other factors have been demonstrated to play a role in healthy ageing. Social isolation and loneliness pose as big of a risk for physical and mental health [24] as other health-related risk factors including smoking, high blood pressure, obesity, and physical inactivity [10]. Additionally, a higher quality and quantity of social relationships have been associated with improved mental health, lower morbidity, and lower mortality [25]. Epidemiological studies have shown that a diet that is high in fruit and vegetable content and lower in dairy and red meat content is beneficial for ageing and cognition [11] and is associated with a reduction in the incidence of chronic conditions and frailty among older people [26]. Smoking has been repeatedly demonstrated to decrease life expectancy, increase the incidence of chronic conditions, and worsen cognitive decline [12]. Similarly, regular consumption of alcohol is associated with accelerated brain ageing [12]. Finally, sleep disruption and sleep loss have been linked to early onset of ageing-related diseases and diminished survival [27,28].

While these healthy ageing behaviours are well established, supporting people in adjusting their lifestyles to include healthy ageing behaviours can be challenging [29]. In an attempt to promote healthy ageing behaviours in communities, ageing centres have been established in several countries, including the Association of South-East Asian Nations (ASEAN) Centre for Active Ageing and Innovation (ACAI) created in Thailand in 2018 and the Older People’s Association (OPA’s) established by HelpAge in multiple countries in Asia [30], as well as centres in the United States [31], Canada [32–34], and Norway [35], among others [36]. However, these countries have different cultural contexts and are experiencing different demographic shifts from Eastern Europe and Central Asia [2]. In the Eastern European context, BiH was among the first to introduce ageing centres to help promote healthy ageing, making it an important example for exploring the applicability of these approaches in Eastern Europe and Central Asia.

In BiH, the non-governmental organisation (NGO) Partnership for Public Health (PPH) has established 17 Healthy Ageing Centres (HACs) in nine municipalities across the country to support older people, with any person over the age of 50 being eligible to join any centre and participate in any of the activities. These HACs provide a series of creative, social, recreational, and educational activities for older people, including daily exercise classes, with the intent of fostering a social environment which promotes healthy ageing behaviours. This cross-sectional study aims to explore the extent to which participation in these centres in BiH is associated with healthy ageing behaviours. This study hypothesises that members of HACs will have an increase in healthy ageing behaviours in comparison to non-members.

## 2 Materials and Methods

### 2.1 Data and Participant Recruitment

This study performed analyses on a subset of secondary data from surveys that were collected in BiH by the NGO PPH. This survey took place between November and December 2019. Four hundred and fifty-four participants were recruited across five HACs in Banja Luka and Sarajevo. The survey was conducted in the local language and consisted of 87 questions, based on the survey in the UK Biobank study [37]. The questions surveyed demographic information, health, and a variety of health behaviours including physical activity, social interactions, diet, alcohol consumption, and smoking, among others, based on activities that are thought to affect healthy ageing [37]. These survey questions have been validated in this demographic group [37].

Six trained individuals from the PPH administered the survey verbally in person and recorded the responses electronically. In-person survey administration was chosen as it has been demonstrated to increase self-report accuracy in older people [38], as well as reduce bias in participants’ education and health status that is associated with telephone and online surveys in older people [39,40]. Because the study was exploratory and due to the difficulties in conducting longitudinal surveys in older populations due to high dropout rates [41], a cross-sectional survey was used for this analysis. The study was based on self-report answers from participants, which were recorded in one session, lasting an average of 16 min. Each question required participants to select the best answer from a list of options. In all cases, there was an option to select that none of the answers applied or that the participant preferred not to answer. Data from the participants were anonymised upon completion of the survey.

Due to the exploratory nature of this study and the importance of including HAC members in the sample, who are only a fraction of the general population, convenience sampling was employed [42]. All people who were in attendance at the HAC on the days in which the survey was being conducted were asked if they wanted to participate in the survey upon arrival. Each HAC had at least two days in which the survey was available. A total of 399 individuals who attended HACs at least once per week were recruited and defined as HAC members, and 55 individuals who attended HACs zero days per week were recruited and defined as non-members. This group of non-members arrived at one of the HACs during the survey period with an interest in becoming an HAC member, but they had not attended any HACs in the past.

### 2.2 Measures

This study focused on several questions on healthy ageing behaviours from the survey, including those about lifestyle habits, physical activity, social interactions, employment status, and life satisfaction. In some cases, multiple questions were combined to create new variables. The survey questions are listed in Table 1.

The answers related to physical activity were combined to calculate the total MET hours per week, based on established MET value calculations for populations over 60 years old [43,44]. Based on the reported exercise measures in the survey, MET hours per week was calculated: (2.5 × minutes walked per day × days walked per week) + (3.0 × minutes of moderate exercise per day × days of moderate exercise per week) + (8.0 × flights of stairs climbed per day × 7 days per week). Family relationship satisfaction and friendship relationship satisfaction were also combined to create a new variable which encompassed general relationship satisfaction. This was calculated by taking the highest absolute value response from the two questions.

### 2.3 Statistics

The results from the survey were analysed using MATLAB (R2024a). The response data for each question were separated into HAC members and non-members as described above. The Shapiro–Wilk test was used for each measure to test for normality [45]. None of the data formed a normal distribution, so tests for non-normal distributions were used [46–48]. A Chi-squared test for categorical measures [48] and a Wilcoxon rank sum test for numerical measures [47] were performed to determine whether there was a significant difference between the HAC member and non-member groups. These two tests are appropriate for unequal sample sizes between groups [48,49].

For categorical variables, the percentage of the population that answered yes to each option was reported. These variables included employment status (employed, unemployed, retired, homemaker, voluntary worker, or unable to work), smoker, gender, attendance at social activities, and loneliness. A bootstrapping approach was used to sample the data with replacement 1000 times to create 95% confidence intervals for categorical variables, with each member and non-member distribution being sampled separately [50,51].

The numerical variables were reported as means with standard deviation (SD) or medians with interquartile ranges (IQR). These variables included age, servings of fruit and vegetables consumed per day, glasses of water consumed per day, servings of meat consumed per day, glasses of alcohol consumed per week, hours of sleep per 24 h, minutes of moderate activity per day, MET hours per week, health satisfaction, relationship satisfaction, financial satisfaction, and work/activity satisfaction.

The bootstrapped distributions [50,51] for the members and non-members were used to calculate the mean effect size (mean of the HAC member group—mean of the non-member group) with 95% confidence intervals for each variable. A series of multiple linear regression models [52] were developed, one for each dependent variable: METs, participation in social activities, health satisfaction, relationship satisfaction, financial satisfaction, and work/activity satisfaction. In each model, HAC attendance per week was an independent variable, with adjusted variables including gender and age. Regression coefficients and standard errors for HAC attendance are reported for each model. For all measures, statistical significance was set at *p* < 0.05.

## 3 Results

### 3.1 Demographic Variables

HAC members attended the centres a median of four days per week, with 42.4% of respondents attending five days per week. Non-members attended zero days per week by definition. HAC members had been attending the HACs for a mean of 21 months prior to this study, with the longest-standing members attending for the previous 96 months. Table 2 reports the demographics of HAC members and non-members. HAC members were older than non-members. Gender differences between the groups were significant, with a higher proportion of females in the member group compared with the non-member group. Most of the participants were retired, with a higher proportion of non-members being a homemaker, unable to work, or unemployed. There were no significant differences between members and non-members in lifestyle habits, including smoking, servings of fruit and vegetables consumed per day, glasses of water consumed per day, servings of meat consumed per day, and glasses of alcoholic drinks consumed per week. HAC members reported sleeping fewer hours per day than their non-member counterparts.

### 3.2 Life Satisfaction Variables

The category of life satisfaction variables included four domains: health satisfaction, relationship satisfaction, financial satisfaction, and satisfaction with work/activities. Health satisfaction was significantly higher for HAC members than non-members (Table 3). Relationship satisfaction with family and friends was also significantly higher for HAC members than non-members (Table 3). Financial satisfaction was significantly higher for HAC members than non-members, and work/activity satisfaction, which may reflect some activities at HACs, was also significantly higher for HAC members relative to non-members (Table 3). Overall, HAC members reported higher satisfaction levels in all domains of life satisfaction than non-members. When adjusted for gender and age in a multiple linear regression model, HAC attendance per week was significantly positively associated with health satisfaction, relationship satisfaction, financial satisfaction, and work/activity satisfaction (Table 4).

### 3.3 Physical Activity Variables

Physical activity variables included the total minutes of moderate activity per day, the percentage of people who exceeded the recommended 150 min of physical activity per week [53], and total MET hours per week. HAC members take part in moderate exercise and activities for a longer time per day than non-members (Table 3). There was no significant difference between the proportion of members and non-members who exceed the public health recommendations of at least 150 min of moderate exercise per week; however, HAC members have 22.75 MET hours per week on average, which is significantly higher than non-members at 18.13 MET hours per week (Table 3, See Section 4). When adjusted for demographics including gender and age in a multiple linear regression model, HAC attendance per week was significantly positively associated with MET hours per week (Table 4). Note that differences in METs between HAC members and non-members exceeds that expected based solely on the observed differences in moderate exercise per week (expected difference of 264 MET minutes for moderate exercise alone versus the observed difference of 400 MET minutes for total METs).

### 3.4 Social Interaction Variables

Social interaction variables include the fraction of people who attend social activities and the fraction of people who say that they are lonely. HAC members are significantly more likely to attend social activities, with ninety-eight percent saying they attend social activities compared to seven percent of non-members, who are less likely to respond that they are involved in any type of social activity including social gatherings, religious gatherings, sports groups, education groups, or other social groups (Table 3). When adjusted for gender and age in a multiple linear regression model, HAC attendance per week was significantly positively associated with participation in social activities (Table 4). Despite high attendance rates at social activities within the HAC member group, loneliness reports are high among all participants in the study. Seventy-five percent of members and eighty-seven percent of non-members reported that they are lonely (Table 3), with no significant difference between the two groups.

## 4 Discussion

This study examines if attendance at HACs is associated with increased healthy ageing behaviours among a select group of older people in BiH. The results support the hypothesis, with HAC members engaging in physical activity for longer time periods and having higher levels of social engagement, both of which are featured activities at HACs. HAC members also report having a higher satisfaction across four areas of their lives. These results are consistent with other studies in the literature which demonstrate that a social network, like those provided by these centres, promotes healthy behaviours in older populations [54,55] and that attendance at ageing centres globally across Western Europe, North America, and Asia [36] is associated with positive health outcomes. This includes higher quality of life [31,56,57], consistent with the results in BiH, and improved mental health [58–60]; however, the effects of ageing centre attendance on physical activity have been mixed [31,36,56,61]. These consistencies and differences from the results observed in BiH are discussed below.

### 4.1 HAC Members Have a Higher Life Satisfaction than Non-Members

HAC members reported higher life satisfaction across health, relationships, finances, and work/activities. Van Leeuwen et al. [62] have suggested that these measures of life satisfaction may be a good proxy for measuring quality of life (QoL) [62], which has become the central focus of ageing initiatives by the World Health Organization (WHO) [63] and the United Nations Economic Commission for Europe [64]. QoL can be a good measure of well-being and a predictor of longevity [65], and focusing ageing interventions on increasing QoL years, rather than just life expectancy, can reduce disability, as well as healthcare costs [66]. The results in BiH are consistent with studies in a number of countries, which showed that attendance at ageing centres and participation in activities at these centres was associated with a higher quality of life and/or life satisfaction [31,56], including among older people who are living with disabilities or are frail [57].

### 4.2 HAC Members Are More Physically Active than Non-Members

HAC members had higher levels of moderate physical activity per week and higher MET hours per week than non-members. Increasing physical activity improves well-being and cognition in older people [67], as well as reduces the risk of dementia [68] and the overall risk of mortality [14]. Even though engaging in physical activity is one of the most impactful interventions to enhance health [69], physical activity is known to decline with age [70]. Adults over 50 years of age remain the most sedentary group of the population [71]. The prevalence of physical inactivity increases globally with age [72], with evidence suggesting that up to 29% of populations over 55 years of age in Europe are physically inactive [73].

The WHO recommends 150 min of moderate exercise per week for the population over 65 years of age [53]. The average weekly exercise of both groups in this study surpassed this recommendation. Given the range of intensity of exercise among older people, MET hours per week provide a more nuanced measure of physical activity. While the median MET hours for both HAC members and non-members surpassed the minimally active threshold, HAC members have significantly higher MET hours per week than non-members, suggesting that HAC members may acquire further health benefits from the physical activity undertaken [74,75]. The exact benefits of these increases in METs are hard to predict, but one study has indicated that an increase in METs equivalent to that observed for HAC members is associated with a projected increase in life expectancy of 0.3 years [75].

In the literature, the relationship between attendance at ageing centres and physical activity levels has been mixed, with some studies finding attendance to be associated with increased physical activity [56], as was observed in BiH, and other studies seeing no effect [31] or effects specific to subpopulations, including women or younger populations [61]. One straightforward explanation for the increased physical activity results in BiH is that more women attend HACs (Table 2), and women are more likely to increase physical activity levels as a result of attending an ageing centre [61]. Alternatively, social support has previously been demonstrated to increase adherence to exercise programs in older adults [76]. Therefore, another possible explanation for the differences in the outcomes of these studies is that the social networks are stronger in some ageing centres than others, with the HACs in BiH providing stronger social networks that help promote increased member engagement in physical activity. While this hypothesis needs to be directly tested, incorporating social components to exercise programmes at ageing centres may help increase consistent physical activity in older populations.

### 4.3 HAC Members Engage in More Social Activities than Non-Members

A diverse social network enhances mental health, general health, and longevity [25,77]. It also improves a sense of purpose in life [78], which has been shown to improve cognition and physical function, while reducing the risk of ageing-related diseases [79,80]. In contrast, social isolation and loneliness are proposed to have comparable effects on mortality to other established risk factors including smoking [81]. Loneliness increases the risk of dementia [82], coronary heart disease [83], and all-cause mortality [84], making it a global health risk.

Loneliness risk often increases during significant life transitions, including retirement, decline in health, and loss of family and friends [85], making it particularly relevant for older populations. These life transitions make older people more susceptible to reduced and less diverse social networks [86], meaning it is essential to promote and support social relationships to limit isolation and prevent loneliness [87]. Even though nearly all HAC members in this survey report regularly taking part in social activities as a part of a social group, reports of loneliness are high among all participants in this study, suggesting that social engagement alone is not sufficient to reduce loneliness. These results are consistent with those of other studies, showing that informal or unstructured social activities are less effective at reducing loneliness levels [88,89], as many older people experience maladaptive social cognition and have social anxiety surrounding social engagement without a common goal. Offering activities that are not just social but have a purpose, like volunteering, can help address this. Furthermore, interventions addressing social confidence that help build social skills or provide one-to-one mental health support to address negative social mindsets have been successful in addressing loneliness, but they can be time- and cost-intensive [88,89]. Group mental health programmes can be more cost-effective and successful than one-to-one support, as the group infrastructure supports the person beyond the duration of the programme [90,91]. Thus, group mental health programmes that address social confidence skills may provide a future approach to help address loneliness, although the efficacy of these types of programmes would need to be directly tested.

### 4.4 Limitations

This exploratory study provides a starting point for building evidence in support of the establishment of HACs on a wider scale; however, there are a number of limitations of this cross-sectional study that uses convenience sampling and self-report surveys.

In order to ensure participation of HAC members, convenience sampling was used at the HACs. This approach has the limitation that the participant sample (both HAC members and non-members) is biased towards older people living in urban areas, where HACs are located, as well as those who are physically fit to attend. Therefore, the generalisability of these results to individuals who live in rural regions or have mobility restrictions is limited due to this bias. There is also a potential selection bias resulting from the recruitment of participants at HACs on a series of random days. This sample may be biased towards participants that attend daily and therefore engage in the centres’ activities most frequently. Thus, the healthy activities that the average HAC member takes part in at the HACs may be lower than what is reported here. This convenience sampling approach also resulted in an unequal sample size in this study, which limits the statistical power of the analyses. While the statistics used in this study account for unequal sample sizes and bootstrapping was used to report confidence intervals on the effect sizes, there is still a risk of type I errors in the results [92].

The use of self-reported data from surveys is another limitation of this study. Self-report introduces the risk of social desirability bias and inaccurate recall [38]. These two biases are known to play a role in a number of the topics addressed in this survey, including loneliness [88], well-being [93], dietary reports [94], exercise time [95] and sleep [96]. In all of these cases, the absolute levels reported may not be accurate, although this would likely be the case for both HAC members and non-members. Furthermore, in older populations, self-report questions that have multiple response options often show a response bias towards later options in the list [38], which tended to be more positive in this survey. Again, this bias would likely affect both HAC members and non-members.

This cross-sectional study is also limited in the interpretation of the directionality of the correlations and potential causality. Future studies should aim to assess the impact of these centres on healthy behaviours longitudinally over time, preferably tracking participants before and after they join the HACs, to establish the long-term impact of participation. Longitudinal studies could help address the question of directionality of the correlations between HAC attendance and healthy behaviours, which currently remains unclear. It is possible that these centres attract individuals who have a higher life satisfaction and engage in more physical and social activity to begin with. Whether HACs actively promote these behaviours or simply provide an outlet for people to engage in these healthy activities is not clear, but in either case, their implementation could still be beneficial for at least a subset of the older population by providing a place to support these behaviours. It is also not clear whether the non-members serving as the control group in this survey went on to become HAC members and, if they did, how active they are at the HAC. Collecting this information in the future could help establish the demographics and characteristics of people who do and do not join HACs, which could inform future recruitment and activity development for HACs to attract a wider range of members.

### 4.5 Implications

This study focuses on HACs in BiH, a country where there is already significant population ageing. Currently, 18% of the population is over the age of 65 years old, with projections for this to increase to over 30% by 2050 [2]. BiH has one of the lowest birth rates in the world at 1.3 children per woman and a high life expectancy at 77.9 years old. Historically, they have also had high net outward migration rates of working age individuals [97], all of which contributes to an ageing population and a limited number of local family members to help support older people. Previous studies have demonstrated an important role for ageing centres in supporting the health of older people who do not have family or carers nearby [56,98], which may be relevant in BiH. The results from BiH may generalise to other countries with high levels of migration of young workers, which includes neighbouring countries in Eastern Europe and Central Asia, many of which have similar demographics to BiH [2]. Thus, the expansion of HACs into other countries in the region may see benefits for the older population.

These centres may also play an important role in supporting clinical practice by providing community-based support for older people. Previous studies have indicated that people with mental health conditions requiring medical care have better long-term outcomes if they also have community support networks [91], like those provided by HACs. This enhanced well-being may also result in better responses to clinical interventions, including better postoperative outcomes following surgery [99]. The implementation of HACs more widely would also move towards preventative healthy ageing approaches rather than only disease management. Preventative approaches may have social, health, and financial benefits [100,101], although the details are debated [102]. In theory, the HACs may provide community support for older people and help identify individuals who need referrals to medical care that they may not seek themselves. In practice, these centres likely target a subset of society that is healthier and are less likely to attract the members of society at disproportionate risk of health inequities. For those that do attend HACs, attendance is associated with higher life satisfaction, physical activity, and social engagement, all of which have been linked to improved health outcomes, including the delay and prevention of ageing-related diseases.

### 4.6 Conclusions

Overall, the present study provides evidence for the association between HAC attendance and healthy ageing behaviours, such as higher life satisfaction, physical activity, and social engagement, among a subset of the older population in BiH. These results are consistent with previous studies globally and provide evidence that the implementation of HACs may be beneficial for older people in the Eastern Europe and Central Asia region.

## Figures and Tables

**Table 1:** Questions from the survey used in this study.

Question	Potential Answers
What is your age?	Number
What is your gender?	Male/Female
Which of the following describes your work situation? You can choose more than one: employed, unemployed, retired, homemaker, voluntary worker, or unable to work.	Any combination of employed, unemployed, retired, homemaker, voluntary worker, unable to work, or none
Do you smoke tobacco now?	Yes/No
How many servings of fruit and vegetables do you have on average per day?	Number up to 10
How many servings of meat do you have on average per day?	Number up to 5
How many glasses of water do you drink on average per day?	Number up to 10
How many glasses of alcoholic drinks do you drink on average per week?	Number
How many hours do you sleep every 24 h?	Number up to 12
In general, how satisfied are you with your health?	Scale 1–6; one being extremely unsatisfied and six being extremely satisfied.
In general, how satisfied are you with your family relationships?	Scale 1–6; one being extremely unsatisfied and six being extremely satisfied.
In general, how satisfied are you with your friendships?	Scale 1–6; one being extremely unsatisfied and six being extremely satisfied.
In general, how satisfied are you with your financial situation?	Scale 1–6; one being extremely unsatisfied and six being extremely satisfied.
In general, how satisfied are you with your work/activities?	Scale 1–6; one being extremely unsatisfied and six being extremely satisfied.
In the past 4 weeks, did you spend any time doing exercise?	Yes/No
How many minutes do you spend doing moderate activity per day?	Number
How many days per week do you do moderate activity for at least 10 min?	Number 0–7
How many minutes do you spend walking per day?	Number
How many days a week do you walk for at least 10 min?	Number 0–7
Over the past 4 weeks, on average, how many times a day do you climb a flight of stairs?	Number
Do you attend social activities at least once a week (including HACs, religious gatherings, sports clubs, education groups, and other social groups)?	Yes/No
If you attend HACs, how many days per week do you attend on average?	Number 0–5
If you attend HACs, for how many months have you been attending?	Number
Do you often feel lonely?	Yes/No

Questions from the survey used in this study, along with the potential answers for each question.

**Table 2:** Demographic information for HAC members and non-members.

Variable	HAC Members [n = 399]	Non-Members [n = 55]	*p*-Value	Effect Size
	% of HAC members (CI) [n_r_]	% of Non-members (CI) [n_r_]	Chi-squared	Mean (CI)
**Gender (female)**	**76.4 (72.0, 80.5) [399]**	**58.2 (45.5, 70.9) [55]**	**0.004**	**18 (6, 32)**
Employment status:				
Employed	1.0 (0.3, 2.3) [399]	1.8 (0,10.9) [55]	0.587	–1.0 (–5, 2)
**Unemployed**	**0.5 (0,1.8)**	**1.8 (0, 9.1)**	**0.026**	–**1.0 (**–**5,1)**
**Retired**	**98.0 (96.2, 99.0)**	**89.1 (78.2, 94.6)**	**<0.001**	**9.0 (1, 18)**
**Homemaker**	**10.8 (8.1, 14.3)**	**29.1 (17.5, 41.8)**	**<0.001**	–**18.0 (**–**31, **–**6)**
Voluntary work	2.8 (1.5, 4.8)	3.6 (0, 12.7)	0.714	–1.0 (–7, 3)
**Unable to work**	**0.5 (0, 1.5)**	**5.5 (1.8, 12.7)**	**<0.001**	–**5.0 (**–**12,1)**
Smoker	17.8 (13.0, 23.3) [399]	30.9 (16.4, 52.7) [55]	0.14	–13.1 (–32, 5)
	Mean (SD) [n_r_]	Mean (SD) [n_r_]	Wilcoxon rank sum	
Lifestyle habits:				
Fruit and vegetables—servings per day	2.33 (1.32) [399]	2.02 (0.95) [55]	0.077	0.31 (0.02, 0.58)
Water—glasses per day	4.76 (2.20) [399]	4.64 (2.90) [55]	0.752	0.12 (–0.66, 0.84)
Meat—servings per day	0.76 (0.81) [399]	0.82 (0.77) [55]	0.512	–0.05 (–0.29, 0.17)
Alcohol—glasses per week	0.41 (1.08) [399]	0.82 (1.97) [55]	0.113	–0.41 (–1.0, 0.12)
**Sleep—hours slept per 24 h**	**4.95 (3.19) [399]**	**5.96 (2.65) [55]**	**0.028**	–**1.0 (**–**1.75, **–**0.24)**
**Age**	**73.14 (6.74) [399]**	**70.22 (7.68) [55]**	**0.004**	**2.95 (0.78, 5.14)**

HAC member and non-member demographics, employment status, and lifestyle habits. A Chi-squared and Wilcoxon rank sum test is used as noted, and respective *p*-values are reported. The data are bootstrapped, and the mean effect size (members—non-members) and 95% confidence intervals are reported from the bootstrapped distributions. Percentages reflect the percentage of respondents who answered yes to the prompt, with 95% confidence intervals calculated from bootstrapped data. SD, standard deviation. CI, 95% confidence intervals. n, the total number of participants. n_r_, number of participants who responded to each prompt. Statistically significant (*p* < 0.05) results are reported in bold.

**Table 3:** Life satisfaction and healthy ageing behaviours for HAC members and non-members.

Variable Theme	Variable	HAC Members [n = 399]	Non-Members [n = 55]	*p*-Value	Effect Size
		Mean (SD) [n_r_]	Mean (SD) [n_r_]	Wilcoxon rank sum	Mean (CI)
	**Health satisfaction (1–6)**	**4.35 (0.82) [398]**	**3.98 (0.73) [55]**	**0.002**	**0.37 (0.16, 0.59)**
**Life satisfaction variables**	**Relationship satisfaction (1–6)**	**4.83 (0.79) [398]**	**4.53 (0.57) [55]**	**0.003**	**0.23 (0.07, 0.40)**
	**Financial satisfaction (1–6)**	**3.88 (1.07) [397]**	**3.53 (0.81) [55]**	**0.003**	**0.36 (0.12, 0.60)**
	**Work/activity satisfaction (1–6)**	**4.54 (0.79) [399]**	**4.16 (0.60) [55]**	**<0.001**	**0.38 (0.20, 0.56)**
		Median (IQR) [n_r_]	Median (IQR) [n_r_]	Wilcoxon rank sum	
**Physical activity variables**	**Moderate activity mins /day**	**30 (15, 60) [399]**	**30 (2.5, 30) [55]**	**<0.001**	**12.58 (6.01, 19.33)**
	**MET hours/week**	**22.75 (12.5, 34.6) [397]**	**18.13 (8.88, 25.6) [55]**	**<0.001**	**6.67 (1.18, 11.81)**
		% of HAC members (CI) [n_r_]	% of Non-members (CI) [n_r_]	Chi-squared	
**Physical activity variables**	Exceeds public health recommendations of >150 min per week	49.4 (44.5, 54.3) [399]	47.3 (34.1, 60.5) [55]	0.77	2.35 (0.18, 6.78)
**Social interaction**	**Participation in social activities**	**98.5 (97.0, 99.5) [399]**	**7.3 (1.8, 16.4) [55]**	**<0.001**	**91 (84, 97)**
**variables**	Loneliness	75.2 (70.66, 79.08) [392]	87.3 (75.47, 94.34) [53]	0.537	12 (2, 22)

Satisfaction scores range from 1 (extremely unsatisfied) to 6 (extremely satisfied). A Chi-squared and Wilcoxon rank sum test is used as noted, and respective *p*-values are reported. The data are bootstrapped, and the mean effect size (members—non-members) and 95% confidence intervals are reported from the bootstrapped distributions. Percentages reflect the percentage of respondents who answered yes to the prompt with 95% confidence intervals calculated from bootstrapped data. SD, standard deviation. n, the total number of participants. CI, 95% confidence intervals. n_r_, number of participants who responded to each prompt. IQR, interquartile range. Statistically significant (*p* < 0.05) results are reported in bold.

**Table 4:** Multiple linear regression models adjusted for age and gender.

Variable	METs	Participation in Social Activities	Health Satisfaction	Relationship Satisfaction	Financial Satisfaction	Work/Activity Satisfaction
HAC attendance						
β (SE)	**3.14 (0.51)**	**0.12 (0.006)**	**0.11 (0.02)**	**0.09 (0.02)**	**0.06 (0.03)**	**0.12 (0.02)**
*p*-value	***p*< 0.001**	***p*< 0.001**	***p*< 0.001**	***p*< 0.001**	***p*= 0.03**	***p*< 0.001**

Multiple linear regression models with HAC attendance per week as the independent variable for each of the listed dependent variables. The models are adjusted for age and gender. β, regression coefficient. SE, standard error. Significant associations are bolded (*p* < 0.05).

## Data Availability

Restrictions apply to the availability of these data, which were obtained from the United Nations Population Fund (UNFPA) in Bosnia and Herzegovina for the current study, but the data are available from the authors upon reasonable request with the permission of UNFPA.
